# More about Private Hospitals

**Published:** 1921-02-26

**Authors:** 


### MORE ABOUT PRIVATE HOSPITALS.

It must be obvious that private hospital reform cannot become more than a pious wish, save by process of law. Everybody may not quite realise that the defect lies with the Legislature, and that, however glaring the scandal may be, the persons responsible are strictly within their rights. The press may accomplish much by calling public attention to the matter, but there its power and influence end. In the circumstances it would be invidious and unfair to do more than generalise. To condemn one or even a score of private hospitals on the ground of improper or inadequate equipment, however serious, would be merely to victimise individuals in a particular locality, and could not possibly effect any general improvement. There would probably be acrimonious debate, if not a lawsuit, and, whatever the conclusion, the main issue would remain. An aggrieved patient can accomplish even less in this direction than a newspaper. Unless his case is overwhelmingly convincing he will probably be described as a crank. There is one only way to reform, and this is by an Act of Parliament making it necessary for the proprietors of private hospitals to conform with conditions to be prescribed by the Minister of Health.

### The Protecting Presence.

There is nothing more revolting than trading on the necessity of the sick and suffering, and while one would shrink from classifying, even by accident, the innocent with the guilty, it cannot be denied that a large number of "nursing homes" are run by unscrupulous people whose chief anxiety is to keep within the law. The requirements of the law are simple. If a doctor puts in an appearance the private hospital is in all respects within bounds. The sewerage arrangements may be defective, the roof and the floors in decay, the nurses of the Gamp genus—none of these things count if only there is a doctor. And what is there to prevent a needy doctor from embarking on such an enterprise? It appears to me a safer and more reputable line of business than some degenerate members of an honoured profession are ready to undertake.

### The Urgency of Legislation.

If the evil is to be cured there must be legislation. It should be laid down as an essential condition that premises used for the purposes of a private hospital should be approved by a Health Department Inspector. A second condition should provide that whole-time fully-trained nurses should be employed in numbers regulated by the number of beds. In fixing the nursing strength of an institution regard would naturally be paid to reasonable hours of employment. Then the premises should be licensed and subject to periodic inspection. Such reform is urgently required in the public interest. The tendency at the moment is for private hospitals to increase. The public demand is becoming greater, and it is imperative that proper accommodation and nursing should be available, more especially as there is no question of charity.

IERNE.

